# Topically applied ZnO nanoparticles suppress allergen induced skin inflammation but induce vigorous IgE production in the atopic dermatitis mouse model

**DOI:** 10.1186/s12989-014-0038-4

**Published:** 2014-08-14

**Authors:** Marit Ilves, Jaana Palomäki, Minnamari Vippola, Maili Lehto, Kai Savolainen, Terhi Savinko, Harri Alenius

**Affiliations:** 1 Nanosafety Research Centre, Finnish Institute of Occupational Health, Helsinki 00250, Finland; 2 Department of Materials Science, Tampere University of Technology, Tampere 33101, Finland; 3 Unit of Systems Toxicology, Finnish Institute of Occupational Health, Helsinki 00250, Finland

**Keywords:** ZnO, Metal oxides, Nanoparticles, Inflammation, Atopic dermatitis, Sunscreens

## Abstract

**Background:**

Metal oxide nanoparticles such as ZnO are used in sunscreens as they improve their optical properties against the UV-light that causes dermal damage and skin cancer. However, the hazardous properties of the particles used as UV-filters in the sunscreens and applied to the skin have remained uncharacterized.

**Methods:**

Here we investigated whether different sized ZnO particles would be able to penetrate injured skin and injured allergic skin in the mouse atopic dermatitis model after repeated topical application of ZnO particles. Nano-sized ZnO (nZnO) and bulk-sized ZnO (bZnO) were applied to mechanically damaged mouse skin with or without allergen/superantigen sensitization. Allergen/superantigen sensitization evokes local inflammation and allergy in the skin and is used as a disease model of atopic dermatitis (AD).

**Results:**

Our results demonstrate that only nZnO is able to reach into the deep layers of the allergic skin whereas bZnO stays in the upper layers of both damaged and allergic skin. In addition, both types of particles diminish the local skin inflammation induced in the mouse model of AD; however, nZnO has a higher potential to suppress the local effects. In addition, especially nZnO induces systemic production of IgE antibodies, evidence of allergy promoting adjuvant properties for topically applied nZnO.

**Conclusions:**

These results provide new hazard characterization data about the metal oxide nanoparticles commonly used in cosmetic products and provide new insights into the dermal exposure and hazard assessment of these materials in injured skin.

## Background

Nanotechnology is a rapidly developing area offering new prospects in many industrial sectors. Metal oxides are one of the most abundantly produced types of engineered nanomaterials (ENM) with production volumes of up to thousands of tons every year [1]. TiO_2_ and ZnO are well-known and widely used metal oxides in many diverse products, such as paints, coatings and cosmetics products like sunscreens due to their opacifying, antimicrobial or UV protective properties [2],[3]. Nowadays it is estimated that the total production volume of metal oxides being used in the skin care market sector is about 2000 tons [4]. The increased utilization of these materials stems from the problems associated with the bulk-sized inorganic ingredients which were used in the older generations of products. The poor dispersion quality, opaqueness and comedogenic properties of non-nanoscale TiO_2_ and ZnO have encouraged manufacturers to improve their products by reducing the size of the particles [3],[5]. Therefore sunscreens are today one of the few nanomaterial-containing products to which people are intentionally exposed [1].

Atopic dermatitis (AD) is a chronic inflammatory skin disorder with a pathophysiology involving a complex interaction between genetic and environmental factors [6]. It has an increasing prevalence, now affecting as many as 2-10% of adults and up to 30% of children in industrialized countries [7]. AD is highly pruritic relapsing disease [8],[9], that causes psychosocial distress and decreases significantly the quality of life of patients and their families [10]–[12]. Furthermore, AD may progress to allergic rhinitis and asthma over time, in a process called the atopic march [13].

The pathomechanisms underpinning this inflammatory skin disease are complex. The epidermal barrier dysfunction in AD is reported to be related to loss-of-function mutations in the gene encoding (pro-)filaggrin which increases the transepidermal water loss [9] and allows the passive transfer of protein allergens [14]. A common feature of AD is the class switch from Th2 type immunity in the acute phase toward Th1 during the chronic phase [15]. In the acute phase of the disease, infiltration of T cells and eosinophils [8] into the dermis can be observed in parallel with release of Th2 type cytokines IL-4 and IL-13 [16]. In contrast to the Th2 type environment present in acute phase, Th1 cytokines such as IFN-γ predominate in chronic skin lesions [15]. A deficiency in the expression of antimicrobial peptides in inflamed skin of AD patients contributes to the increased susceptibility towards bacterial colonization of the skin [8]. In particular, *Staphylococcus aureus* has been detected on the skin of >90% of AD patients [17]. These bacteria release exotoxins such as staphylococcal enterotoxin B (SEB) that act as a superantigen, inducing T-cell activation and triggering the release of pro-inflammatory, Th2 and Th1 type cytokines, thus aggravating and exacerbating the disease [18]–[20].

Topical exposure to ENM, especially to TiO_2_ and ZnO, and the penetration of the particles into the skin has been investigated previously; however, the results of the studies are somewhat controversial. Furthermore, there is a lack of knowledge about the effects of these materials on injured or diseased skin. When taking into account the high prevalence of AD especially among children, there is some concern about whether ENM can cause health effects if the skin barrier integrity has been is damaged. The aim of this study was to investigate the effects caused by topically applied nano-sized ZnO (nZnO) in the mouse model of AD and to compare these outcomes to those induced by bulk-sized ZnO (bZnO) to better understand the importance of ZnO particle size.

## Results

### nZnO Particles penetrate through the sensitized skin in the mouse model of atopic dermatitis

**Figure 1:**
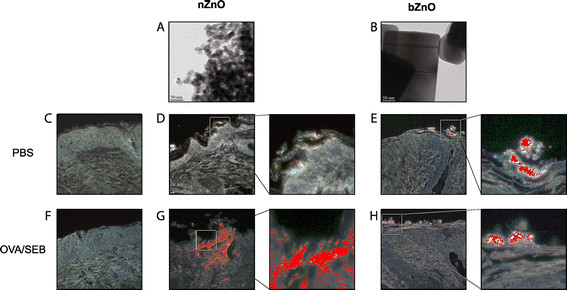
**The morphology of nZnO (A) and bZnO (B) particles by TEM and their translocation into the skin after topical application.** TEM images of nZnO and bZnO materials reveal the differences in particle size and morphology. Scale bar 50 nm. Hyperspectral image analysis of PBS **(C)**, nZnO **(D)**, bZnO **(E)**, OVA/SEB sensitized **(F)**, OVA/SEB and nZnO treated **(G)**, and OVA/SEB and bZnO treated **(H)** skin sites. Images are shown at 40x magnification, insets refer to the presence of particles in the skin or on its surface.

It has been postulated that particles are unable to pass through intact skin but their ability to penetrate into the damaged skin is unknown. The murine model of AD (Additional file 1) was used to study the particle penetration. In this model, a standardized skin injury is caused by tape stripping thus mimicking the frequent scratching which is commonly experienced by AD patients. Skin samples were collected to investigate whether different sized particles could be found in the different skin layers in non-sensitized and OVA/SEB-sensitized mice. Both materials were found in agglomerates on the skin surface. The particles of nZnO, in contrast to bZnO, had a tendency to accumulate more into hair follicles. Furthermore, the presence of nZnO particles was detected in the epidermal and dermal layers of the skin of both PBS-treated and OVA/SEB-challenged mice (Figure 1). Unlike in the skin of vehicle-treated mice, the number of spectral matches of nZnO in epidermis and dermis of AD-like skin lesions (*i.e.* after OVA/SEB challenge) was remarkable, revealing particle accumulation especially in epidermal layer. In contrast to nZnO, bZnO was only detected on the surface of both PBS-treated and OVA/SEB-challenged skin and no particle penetration was detected into the epidermis and dermis. This analysis suggests that AD-like skin enables at least partial penetration of nZnO particles through the damaged epidermis into the viable layers of the skin.

### nZnO Treatment significantly reduces skin thickness in the mouse model of atopic dermatitis

**Figure 2:**
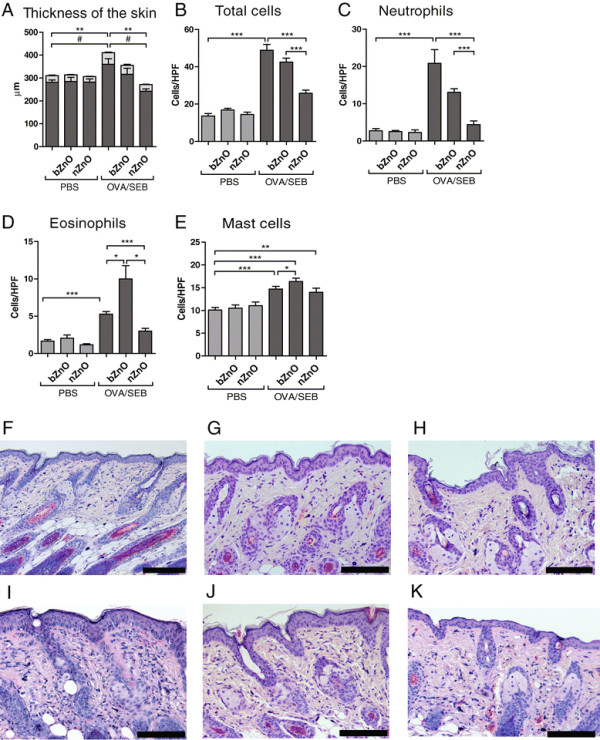
**Thickness of epidermis and dermis, and the number of infiltrating cells on treated skin sites. A**. Measurements were performed from H&E-stained skin samples at 100x magnification. The light gray section in the columns represents epidermal and dark grey is the dermal thickness. Statistical significances are indicated as * for epidermal thickness and # for whole skin thickness. **P < 0.01, #*P* < 0.05. The total inflammatory cells **(B)**, neutrophils **(C)**, eosinophils **(D)** and mast cells **(E)** were counted after H&E at 1000x **(B-D)** and toluidine blue at 400x **(E)** and expressed as cells per high power field (HPF). The columns and error bars represent means ± SEM (n = 8-16 mice per group). H&E-stained skin of PBS **(F)**, bZnO **(G)**, nZnO **(H)**, OVA/SEB sensitized **(I)**, OVA/SEB and bZnO treated **(J)**, and OVA/SEB and nZnO treated skin **(K)**. Scale bar 100 μm. *P < 0.05, **P < 0.01, ***P < 0.001.

Patients with AD have an increased skin thickness and inflammatory cell infiltratration into the inflamed skin lesions. In the non-sensitized skin sites, the treatment with ZnO particles did not cause any changes in the thickness of skin layers whereas in the OVA/SEB-sensitized skin, both the epidermal and dermal thicknesses were reduced in ZnO-treated groups (Figure 2A). However, the reduction was statistically significant only in the nZnO treated group compared to vehicle controls. These results clearly demonstrate that nZnO reduced the skin thickness in the allergic environment more efficiently than bZnO.

### nZnO Application significantly suppresses the influx of inflammatory cells in allergic skin

T cells, eosinophils and mast cells are characteristic cell types in AD skin. OVA/SEB sensitization significantly increased infiltration of all inflammatory cells as compared to non-sensitized mice skin (Figure 2B-K). bZnO and nZnO significantly reduced the number of total inflammatory cells and neutrophils in OVA/SEB-sensitized mice, and the numbers of these cells were significantly lower in nZnO-treated mice skin as compared to bZnO (Figure 2B-C). Interestingly, eosinophil numbers were significantly increased in bZnO group compared to non-particle-treated and to nZnO-treated OVA/SEB-sensitized mice (Figure 2D). In contrast, the numbers of eosinophils were significantly reduced in the skin of OVA/SEB-sensitized nZnO mice as compared to OVA/SEB-sensitized mice. Mast cell infiltration was induced in all OVA/SEB-sensitized groups, however, not as extensively in the nZnO-treated mice as in the other groups (Figure 2E). Representative histological features of the AD model after exposure to bZnO or nZnO are shown in Figure 2F-K. These results indicate that nZnO particles can suppress the infiltration of inflammatory cells more efficiently than bZnO particles in the OVA/SEB-sensitized skin.

### ZnO Particles diminish the infiltration of T-cells in sensitized skin but induce the infiltration of macrophages into damaged skin

**Figure 3:**
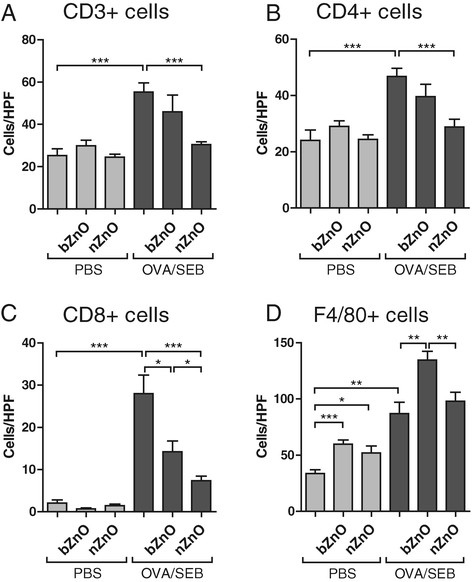
**Distribution of T-cells and macrophages in treated skin samples.** Immunostained DC3^+^**(A)**, CD4^+^**(B)**, CD8^+^**(C)**, and F4/80^+^**(D)** cells were counted at magnification of 400x. The columns and error bars represent means ± SEM (n = 8-16 mice per group). *P < 0.05, **P < 0.01, ***P < 0.001.

Immunohistochemical stainings were performed to analyse different T-cell types and macrophages from sensitized skin sites. The number of CD3^+^, DC4^+^ and CD8^+^ T-cells were significantly induced in the OVA/SEB-sensitized mice skin whereas bZnO and nZnO treatments reduced T-cell numbers in the OVA/SEB-sensitized skin (Figure 3A-C, Additional file 2). Overall T-cell numbers were lower in nZnO-treated mice than in the bZnO group. In addition, macrophage numbers (F4/80^+^ cells) were higher in the skin of particle-treated mice as compared to non-particle-treated mice with an exception of OVA/SEB-challenged and nZnO-treated group where macrophage influx was insignificant in the comparison with the OVA/SEB-sensitized group (Figure 3D, Additional file 3). In the comparison of particle-treated skin samples, it was noted that bZnO induced more extensive macrophage infiltration than nZnO. These results demonstrate that ZnO particles reduce T-cell infiltration in OVA/SEB-sensitized skin. However, macrophages are recruited to the particle-treated tape-stripped skin, independently of the sensitization status.

### mRNA Expression of skin cytokines is downregulated in nzno-treated mice

**Figure 4:**
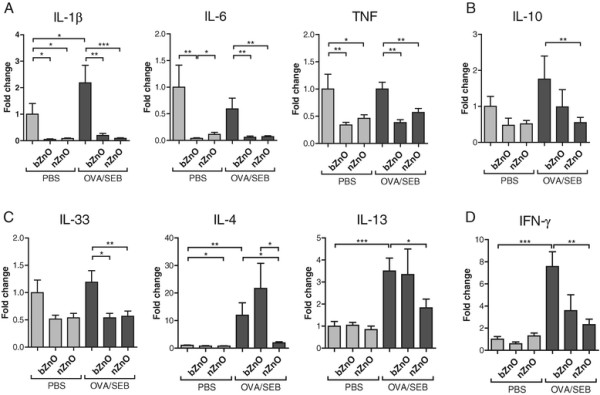
**Fold changes of mRNA levels of pro-inflammatory (A), anti-inflammatory (B), Th2-type (C), and Th1-type (D) cytokines in skin biopsies of treated mice compared with PBS controls.** Cytokine mRNA levels were measured by quantitative real-time PCR and normalized with 18S rRNA. Bars represent means ± SEM (n = 8-16 mice per group). *P < 0.05, **P < 0.01, ***P < 0.001.

Lesional skin of AD patients exhibits increased expression of pro-inflammatory, Th2 and Th1 cytokines [16],[21]. The levels of pro-inflammatory cytokines (IL-1β, IL-6, TNF), anti-inflammatory cytokine (IL-10), Th2-type cytokines associated with allergy (IL-4, IL-13, IL-33) and Th1-type cytokine (IFN-γ) were analysed from the treated skin sites of all groups. Transcription of all studied cytokines was induced by OVA/SEB sensitization. The mRNA levels of pro-inflammatory cytokines were suppressed by ZnO particle treatment (Figure 4A). The same phenomenon was seen in IL-10 and IFN-γ mRNA levels (Figure 4B, D). In addition, the expression of Th2-type cytokines was significantly suppressed by nZnO but not by bZnO (Figure 4C). Cytokine analysis demonstrates that nZnO particles downregulated mRNA expression of different types of cytokines whereas bZnO particle treatment did not suppress Th2 type cytokines in the mouse model of atopic dermatitis.

### nZnO Suppresses the production of IL-13 and IFN-γ cytokines in sensitized mice

**Figure 5:**
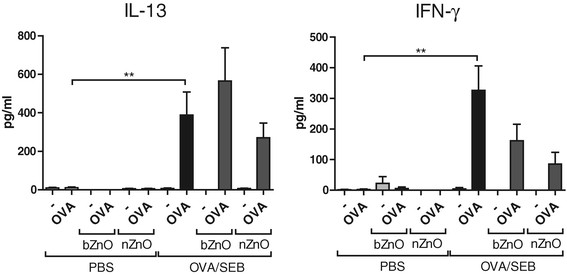
**Secretion of IL-13 and IFN-γ protein in lymph node cells re-stimulated with OVA*in vitro*after repeated topical application of PBS and OVA/SEB with or without particle application.** Cytokine levels were measured with ELISA. Bars represent means ± SEM (n = 4-8 pooled samples per group). **P < 0.01.

Allergen (OVA) induced systemic production of major Th1 and Th2 cytokines was investigated with circulating lymphocytes from skin draining lymph nodes being harvested and stimulated with OVA. Production of IL-13 protein by lymph node cells collected from OVA/SEB-treated mice was strongly induced after OVA stimulation; however, the levels were slightly, albeit not statistically significantly, decreased after nZnO treatment but not in response to bZnO treatment (Figure 5A). In addition, OVA stimulation of cells collected from OVA/SEB-sensitized mice induced IFN-γ protein secretion (Figure 5B). The amount of secreted IFN-γ protein was decreased when mice were treated with ZnO particles and the reduction was greater in response to nZnO application. However, the ZnO-induced decrease in IFN-γ protein did not reach statistical significance. These data suggest that topical treatment of mice with nZnO had slightly suppressed Th1 and Th2 type cytokine secretion also in draining lymph nodes stimulated *in vitro*.

### Local treatment with ZnO particles induces a systemic increase in IgE antibody levels in sensitized mice

**Figure 6:**
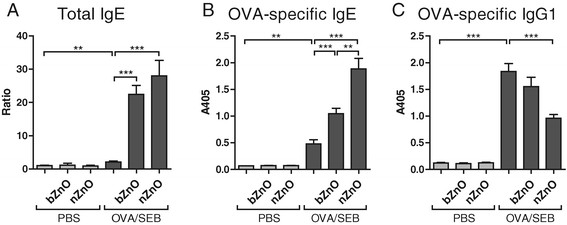
**Antibody levels in mice sera after repeated topical application of vehicle, different sized ZnO particles, and OVA/SEB with or without particle application.A**. Total IgE values are shown as ratios compared to PBS-treated control, **B**. OVA-specific IgE and **C**. OVA-specific IgG1 levels are shown as optical values measured at 405 nm. The columns and error bars represent means ± SEM (n = 8 mice /group). **P < 0.01, ***P < 0.001.

Patients with AD have elevated levels of total and allergen specific IgE antibodies in the serum. In an attempt to evaluate systemic effects of topical ZnO treatment, we measured allergen (OVA)-specific IgG2a (Th1 surrogate), IgE (Th2 surrogate) and IgG1 as well as superantigen (SEB)-specific IgG2 and IgE antibodies from mice sera. Total IgE and OVA-specific IgE levels were induced after ZnO treatment in OVA/SEB-sensitized mice, and nZnO induced the most significantly elevated response (Figure 6A, B). In contrast, allergen induced OVA-specific IgG1 levels in OVA/SEB-challenged mice were significantly reduced after nZnO treatment (Figure 6C). There were no significant differences in total or OVA-specific IgG2a levels between the groups after topical treatment of the mice (Additional file 4). SEB-specific IgG2a or IgE levels did not reveal any differences between treatment groups (data not shown). It is concluded that local treatment with ZnO particles induced a systemic increase in Th2 type antibody, IgE, in the mouse model of AD.

## Discussion

Sunscreens are widely used consumer products that may contain nano-sized inorganic UV filtering agents such as ZnO. The safety of ENM after topical application has been evaluated using both *in vitro* and *in vivo* models but the results emerging from the different studies are controversial. Furthermore, there is a lack of data about whether ENM can penetrate through damaged skin. Due to the increasing prevalence of allergic skin conditions, such as atopic dermatitis (AD), there is a need to understand whether ENM may pose a hazard when applied repeatedly onto injured and diseased skin. This study investigated particle penetration and inflammatory effects of nZnO and bZnO in compromised skin. We used a mouse model of AD where the skin injury is inflicted by tape stripping and the allergic skin condition is triggered by application of two antigens, OVA and SEB. In order to obtain similar dispersion quality and consistency as in sunscreens, ZnO mixtures were prepared in this study with the same nZnO content as allowed in commercial products [22]. Furthermore, we decided to use repeated epicutaneous application of ZnO in our model due to the fact that large numbers of people use sunscreens repeatedly on a daily basis [23].

Previously conducted studies performed on human skin have reported conflicting penetration results after epicutaneous exposure to nZnO. Studies on human volunteers have shown that after a brief exposure to nZnO, there is no penetration not only through viable healthy skin but also through tape-stripped and lesional skin [24]. However, after repeated application of nZnO on healthy human skin, variable amounts of tracer ^68^Zn could be detected in urine and a small amount in blood, evidence of very low absorption of either soluble Zn, ZnO particles or both [23],[25]. The results of our study showed that very small amounts of topically applied nZnO could be detected in the upper layer of epidermis and in openings of hair follicles of tape-stripped non-allergic mouse skin. However, it has been reported that material accumulation in follicular orifices does not pose a risk as the particles remaining there are ultimately removed by sebum flow [26]. Nonetheless, a significant accumulation of nZnO particles in the epidermis and lesser amount also into dermis was detected in the allergic skin. In contrast to the situation with nZnO, bZnO did not penetrate through either mechanically injured or OVA/SEB-treated tissue. These results indicate that inflamed skin of AD patients might experience a notably greater penetration and translocation of nZnO upon repeated application whereas the larger ZnO particles are unable to reach into the deeper layers of the skin.

The thickened skin of AD lesions is accompanied by the recruitment of inflammatory cells. In the acute lesions, the dermal cellular infiltrate consists predominantly of CD4^+^ T cells with some presence of eosinophils and mast cells [27],[28]. This phenomenon was also observed in our study, confirming the functionality of the disease model. Epicutaneous nZnO-treatment resulted in significantly decreased epidermal and dermal thickness and it also diminished dermal neutrophilia in the AD model. Interestingly, exposure to bZnO triggered a significant infiltration of eosinophils into the skin whereas nZnO slightly inhibited their migration. Moreover, the infiltration of macrophages was significantly increased especially in response to topical bZnO treatment. In allergic skin, nZnO significantly inhibited the recruitment of CD4^+^ and CD8^+^ T cells significantly. Taken together, these results demonstrate that allergen induced skin inflammation is significantly reduced, especially by the topical exposure to nZnO particles. In contrast, exposure to bZnO increases the infiltration not only of eosinophils but also of macrophages into AD-like skin lesions.

To further explore the local effects of nZnO and bZnO in the skin, we analysed several of the cytokines that are involved in inflammatory processes of AD. Pro-inflammatory cytokines IL-1β, IL-6 and TNF are released in response to mechanical injury and skin barrier disruption, factors that are associated with scratching in AD [29],[30]. In addition, the anti-inflammatory cytokine IL-10 is known to be overexpressed in skin lesions of patients with AD [31]. Furthermore, recent findings have revealed that the expression of the Th2-promoting cytokine IL-33 is elevated in AD skin after allergen or SEB exposure and thatthis is followed by the production of IL-4 and IL-13 triggering Th2 lymphocyte differentiation [32]. It has also been shown that superantigen exposure in AD skin leads to the production of IFN-γ [19]. In our model of AD, we determined similar cytokine expressions as described in the literature. The expression levels of essentially all cytokines were down-regulated when the mice were treated with nZnO suggesting that the nZnO particles could suppress local inflammation whereas bZnO failed to suppress Th2-type cytokine expression. We also investigated allergen induced cytokine secretion into the draining lymph nodes, in areas distant from the site of the skin inflammation, and found that allergen induced secretion was slightly, albeit not significantly, suppressed, especially by the nZnO. The differences in the local inflammation are probably seen due to the differences in the particle size. As particle size decreases, active surface area and particle reactivity increase [33]. In the water-dissolving ZnO particles, Zn^2+^ ions are released to a greaterextent from nZnO than bZnO [34] and therefore the local effects are stronger if skin is treated with nanoparticles (NP).

Very little is known about the inflammatory effects of ZnO in the context of AD. While nZnO has not been investigated in murine models of AD, the effects of nano-sized TiO_2_ (nTiO_2_) have been described *in vivo*[35]. However, instead of topical application, intradermal administration of low dose of nTiO_2_ was used, assuming that particles would be able to penetrate through the skin. Yanagisawa *et al*. found that nTiO_2_ administration causes aggravation of AD lesions as reflected in elevated levels of Th2-type cytokines in the skin. The results of our study demonstrate that after repeated topical application of nZnO in mice, the material reaches to the dermal layer of AD-like skin but there is no penetration through mechanically injured non-allergic tissue. In contrast to the effects induced by nTiO_2_[35], we found that nZnO could reduce the skin thickness of antigen-sensitized mice and it suppressed cell recruitment to inflammatory site. This was further supported by the inhibition of the pro- and anti-inflammatory, Th2- and Th1-type cytokines in AD-like skin emphasizing the differences in chemical properties of these metal oxide NP. These findings demonstrate that nZnO has a local anti-inflammatory effect on allergic skin.

Patients suffering from AD are known to exhibit elevated serum levels of total IgE and allergen-specific IgE [36]. The present study revealed that total IgE and specific IgE and IgG1 levels in serum were increased in our murine model of AD. While specific IgG1 amounts were reduced in allergic skin upon topical nZnO treatment, the IgE levels, especially the levels of total IgE, were notably enhanced when either of the materials, nZnO or bZnO, were applied onto AD-like skin. The observed effect was greater in response to nZnO treatment than withbZnO. Thus, topical exposure, especially to nZnO, could enhance the secretion of B-cell derived IgE antibodies while it simultaneously dramatically suppresses allergen induced skin inflammation. It has been reported that ZnO NP rapidlyrelease Zn^2+^ ions in phosphate buffer [34]. An earlier study conducted on human volunteers where topical exposure of nZnO was investigated by tracing ^68^Zn in blood and urine, concluded that the detected levels of isotope might have been absorbed as nZnO particles or as Zn^2+^ ions or possibly both [23]. For this reason, there remains the possibility that the elevated IgE levels detected here might be caused by non-specific reactions of released Zn^2+^ ions affecting IgE production capabilities of the B-cells. The higher IgE levels in nZnO-treated as compared to bZnO-exposed AD mice could be explained by the increased release of Zn^2+^ ions from nZnO particles which first accumulated into AD-like skin and ultimately the affected IgE production capacity of the B-cells either in the draining lymph nodes or in the spleen.

The underlying mechanisms of anti-inflammatory (locally in the skin) or adjuvant properties (systemic antibody production) of ZnO are still not fully understood and further studies will beneeded. However, it can be speculated that local concentrations of ZnO particles/Zn^2+^ ions in the skin are optimal for achieving anti-inflammatory effects against allergen induced Th2 type skin inflammation. In contrast, the substantially lower Zn^2+^ ion concentration released into the circulation and further into the draining lymph nodes and the spleen (which are the major sources of antibody production), may be more optimal for enhancing B-cell mediated IgE antibody production. It should be also noted that the cell constituents are very different in the skin compared to the lymphoid organs. Thus, differences in the responding cells as well as in concentrations of ZnO particles/Zn^2+^ ions may elicit very different effects systemically and locally.

## Conclusions

Our results demonstrate that after repeated topical exposure to nZnO, the particles can reach to the dermal skin layer of allergic skin. We also show that nZnO application exerts anti-inflammatory properties by decreasing drastically local skin inflammation in the mouse model of AD. However, clearly elevated systemic production of IgE antibodies in allergic mice was detected in response to nZnO application. Our data suggest that even though exposure of AD patients to nZnO-containing sunscreens could have a beneficial and symptom-relieving effect in the skin, caution is warranted when applying these products onto allergic skin due to the possible aggravation of IgE-antibody secretion. Furthermore, classical sunburns cause mechanical damage and local irritation to the skin, and therefore the skin’s protective barrier against NP may be diminished in the very situation where sunscreens are commonly used [37]. Nevertheless, when taking into account the elevated risk for skin cancers, especially malignant melanoma, without UV-protection, the use of sunscreens may well outweigh the symptoms caused by application of nZnO-containing creams. To conclude, this study provides new information about the effects of nZnO applied onto inflamed skin and in this way contributes to dermal exposure assessment, exposure scenarios and hazard characterization of metal oxide ENM.

## Materials and methods

### Mice and sensitization

Female BALB/c mice (aged 6–8 weeks) were obtained from Scanbur A/S (Karlslunde, Denmark) and quarantined for one week. The mice were housed in groups of four in transparent plastic cages bedded with aspen chip and were provided with standard mouse chow diet (Altromin no. 1314 FORTI, Altromin Spezialfutter GmbH & Co., Germany) and tap water *ad libitum* when not being treated. The environment of the animal room was carefully controlled, with a 12 h dark–light cycle, temperature of 20–21°C, and relative humidity of 40–45%. The experiments were performed in agreement with the European Convention for the Protection of Vertebrate Animals Used for Experimental and Other Scientific Purposes (Strasbourg March 18, 1986, adopted in Finland May 31, 1990). All experiments were approved by the State Provincial Office of Southern Finland.

Mice (eight per group) were epicutaneously treated with vehicle (PBS), bZnO particles, nZnO particles, a combination of ovalbumin and staphylococcal enterotoxin B (OVA/SEB, both purchased from Sigma-Aldrich Co, St. Luis, MO), a combination of OVA, SEB and bZnO or combination of OVA, SEB and nZnO under isoflurane anesthesia (Univentor 400 Anesthesia Unit, Abbott Laboratories, IL). The back of the mice was shaved with an electronic razor and tape-stripped by adhesive tape to induce a standardized skin injury. Stripping included placing a piece of adhering tape onto shaved skin one to four times, after which it was removed against the direction of the hair. The total amount of OVA was 100 μg and SEB 2.5 μg/patch and the mass of bZnO or nZnO particles was 16.67 mg/patch (Additional file 1A). During the first sensitization week, 100 μl of PBS or a mixture of OVA/SEB in PBS was added to sterile gauze and then secured onto the back skin with transparent adhesive tape. After a two-week recovery period, the mice were again tape-stripped and a new patch was secured onto the same skin site. During the second sensitization week, ZnO materials were applied in PBS or OVA/SEB mixture. Patches were applied twice during the first sensitization week and three times during the second sensitization week. Mice were killed by isoflurane overdose 24 h after the last sensitization. Blood samples from the hepatic vein were collected for antibody analysis, skin biopsies from treated skin areas for RNA isolation and histology, and skin draining lymph nodes for re-stimulation with OVA or SEB to analyse OVA and SEB induced secretion of cytokines (Additional file 1B).

### ZnO Particles and suspension preparation

bZnO was ordered from Camden-Grey Essential Oils, Inc (Doral, FL) and nZnO from Nanostructured & Amorphous Materials Inc (Houston, TX). The morphology and size of the materials were characterised by transmission electron microscopy (Jeol JEM 2010 TEM, Jeol Ltd., Tokyo, Japan) as represented in Figure 1 and Additional file 5, respectively. Samples were prepared from the ZnO powders by crushing these gently, dispersing in ethanol and applying a drop of the dispersion onto a copper grid-holey carbon layer sample holder. The accelerating voltage used in TEM was 200 kV. The material composition was determined by energy dispersive spectroscopy EDS (ThermoNoran Vantage, Thermo Scientific, Breda, The Netherlands attached to Jeol JEM 2010 TEM). The elemental composition shown in Additional file 5 is the average of three separate EDS analysis.

ZnO suspensions for experiments were prepared by weighing the materials into glass tubes, dispersing in PBS and water-bath sonicating for 20 minutes at 30°C. Before application onto the patches, the suspensions were diluted to 16.67 mg/ml in vehicle (PBS) or a mixture of OVA and SEB in PBS. The dispersions were mixed before their application onto the gauze patches.

### Analysis of the translocation of ZnO particles in the skin

The CytoViva hyperspectral imaging microscopy system (CytoViva, Inc, Auburn, AL) was used to analyse whether bZnO and nZnO particles could penetrate into the skin in the AD mouse model,. The essential parts of the hyperspectral imaging technology are a transmission diffraction grating spectrograph and an integrated CCD camera that are mounted onto the camera (C) mount of an Olympus BX43 microscope (Olympus Corporation, Tokyo, Japan).

Hyperspectral images were captured, processed and analysed in image analysis software CytoViva ENVI 4.8. Sonicated 100 μg/ml ZnO dispersions in sterile PBS were used for creating spectral libraries of the materials. Hyperspectral images of unstained 4-μm skin sections were captured at 40x magnification. Images were normalized and smoothened with the Savitski-Golay Curve Fit Smoothing algorithm using a width of 11 bands. To exclude the possibility for non-specific matches in images of material-exposed tissue, spectral libraries of both ZnO materials were filtered with spectra collected from PBS-treated and OVA/SEB-challenged skin. Thereafter, Spectral Angle Mapper Classification feature was used to find the locations of pixels matching the spectra of bZnO or nZnO in the images of ZnO-treated skin.

### Skin histology and immunohistochemistry

In the histological analysis, skin biopsies were fixed in 10% buffered formalin and embedded in paraffin and then 4-μm skin sections were cut and stained with hematoxylin and eosin (H&E) for cell counts and to measure epidermal or dermal thicknesses, as well as with o-toluidine blue for mast cell counts. In the immunohistochemical analysis, skin specimens were embedded in Tissue-Tek oxacalcitriol compound (Sakura Finetek Europe B.V., Zoeterwoude, The Netherlands) and frozen quickly on dry ice. Staining of different subtypes of T-cells (CD3+, CD4+ and CD8+) was conducted as described earlier [19]. Briefly, the 4-μm skin samples were fixed with cold acetone and stained using the ChemMate (DakoCytomation, Glostrup, Denmark) staining kit. Primary anti-mouse monoclonal antibodies were obtained from BD Pharmingen (San Diego, CA). Macrophages (F4/80^+^) were stained in a similar technique with purified rat anti-mouse F4/80 antibody (clone BM4007S, Acris Antibodies, Herford, Germany). Biotin-conjugated secondary antibodies were obtained from Vector Laboratories Inc. (Burlingame, CA). The sections were examined under light microscopy (Leica DM 4000B, Wetzlar, Germany). Inflammatory cells were counted from 15 high-power fields (HPF) at 1000x magnification, mast cells, T-cell subtypes and macrophages at 400x magnification.

### Cytokine analysis by RT-PCR

Quantitative real-time reverse transcription-PCR analyses were performed as previously described [19] with 7500 Fast Real-Time PCR system (Applied Biosystems, Foster City, CA). Briefly, skin tissues were homogenized with Ultra-Turrax T8 (IKA Labortechnik, Stafen, Germany) in TRIsure® (Bioline GmbH, Luckenwalde, Germany). RNA was isolated and extracted from skin tissues according to TRIsure® instructions. The concentration of RNA was measured with NanoDrop ND-100 Spectophometer (Thermo Scientific, Wilmington, DE) and cDNA was synthesized with High Capacity cDNA Reverse Transcription Kit (Applied Biosystems). PCR primers and probes were obtained from Applied Biosystems as pre-developed reagents. The gene expression between different samples was normalized to the 18S housekeeping gene. Results are shown as fold change in comparison to vehicle-treated mice.

### Cytokine and antibody measurements by ELISA

Collected lymph nodes were mechanically ground and LN cell suspensions from two mice were pooled (four samples per group were analysed). The LN cell suspensions were prepared in RPMI 1640 medium supplemented with 5% fetal bovine serum (FBS), 1% penicillin-streptomycin (PEST), 1% non-essential amino acids (NEAA), 1 mM sodium pyruvate, 10 mM HEPES (all obtained from Invitrogen, Paisley, UK), 1% Ultraglutamine (Lonza, Basel, Switzerland), and 0.05 mM 2-mercaptoethanol (2-ME, Sigma-Aldrich Co, St. Luis, MO). Cells were cultured in medium at 3 × 10^6^ in 24-well plates in the presence of OVA (50 μg/ml) or SEB (1 μg/ml). The cell culture medium was collected after 48 h of stimulation for protein analysis. Mouse IL-13 and IFN-γ (eBioScience, San Diego, CA) ELISAs were performed according to the instructions given by the manufacturer.

Total and specific antibody levels were determined by the ELISA method. The standard BD Pharmingen protocol for sandwich ELISA was used to quantify the total amount of IgE and IgG2a in the sera. Purified mouse IgE (clone R38-2) or IgG2a (clone G155-178) were used as standards. 96-well plates were coated with rat anti-mouse IgE mAb (clone R35-72) or anti-mouse IgG2a mAb (clone R11-89), and bound IgE and IgG2a were detected with biotin-conjugated rat anti-mouse IgE (clone R35-118) or IgG2a (clone R19-15). Streptavidin-HRP and peroxidase substrate reagents were used to generate the color reaction. All reagents were ordered from BD Biosciences (San Jose, CA) except for the peroxidase substrate reagents which were from Kirkegaad & Perry Laboratories (Gaithersburg, MD).

Serum levels of OVA specific IgE, IgG1 and IgG2, and SEB specific IgE and IgG2a levels were measured by the straight ELISA method. Briefly, 96-well plates were coated (50 μl/well) with 100 μg/ml OVA or 1 μg/ml SEB in 0.05 M NaHCO_3_ (pH 9.6) overnight (+4°C). The plates were washed with 0.05% Tween20 in PBS, blocked with 3% bovine serum albumin (BSA) in PBS for 2 h (20°C) and washed again. 100 μl of diluted sera (IgE: 1/60, 1/180, 1/540; IgG1: 1/2000, 1/20000, 1/200000; IgG2a: 1/60, 1/540, 1/1620) in 1% BSA in PBS was added and incubated overnight (+4°C). After washing, 2 μg of biotin-conjugated rat anti-mouse IgE (clone R35-118), IgG1 (clone A85-1) or IgG2a (clone R19-15) in 1% BSA in PBS was added, incubated for 2 h (20°C) and washed. Streptavidin-HRP (1/4000) in 1% BSA dissolved in PBS was added and incubated for 30 min (20°C). After washing, a mix of peroxidase substrate reagents was added and absorbance read at 405 nm with a Labsystems Multiscan MS-spectrophotometer (Thermo Scientific, Wilmington, DE).

### Statistics

All data are presented as means ± standard error, and the difference between groups was analysed with GraphPadPrism 5 software (GraphPad Software, Inc. San Diego, CA) using Student’s t-test or Mann–Whitney U-test. Differences at *P* < 0.05 were considered to be statistically significant.

## Abbreviations

AD: Atopic dermatitis

BSA: Bovine serum albumin

bZnO: Bulk-sized zinc oxide

CD: Cluster of differentiation

cDNA: Complementary deoxyribonucleic acid

ELISA: Enzyme-linked immunosorbent assay

ENM: Engineered nanomaterials

FBS: Fetal bovine serum

H&E: Hematoxylin and eosin

HPF: High power field

IFN: Interferon

Ig: Immunoglobulin

IL: Interleukin

LN: lymph node

mRNA: Messenger ribonucleic acid

NEAA: Non-essential amino acids

nTiO_2_: Nano-sized titanium dioxide

nZnO: Nano-sized zinc oxide

OVA: Ovalbumin

PBS: Phosphate buffered saline

PCR: Polymerase chain reaction

SEB: Staphylococcal enterotoxin B

SEM: Standard error of the mean

TEM: Transmission electron microscopy

Th: T helper

TNF: Tumor necrosis factor (alpha)

## Competing interests

The authors declare they have no competing interests.

## Authors’ contributions

MI, JP, TS and HA designed and MI performed the animal experiments. MI, JP, TS and ML were involved in sample collection and processing. MI and JP carried out restimulations of lymph node cells *in vitro*. MI performed hyperspectral imaging analysis. JP carried out cell counting and measurements of skin thicknesses. MI performed PCR analysis and JP carried out ELISA assays. MI, JP, TS and HA interpreted the results. MV characterized the materials. MI, JP and HA wrote the manuscript, MV, TS and KS contributed to the manuscript by commenting. All co-authors approved the final manuscript.

## Additional files

## Supplementary Material

Additional file 2: **CD3, CD4 and CD8 stained skin of A. OVA/SEB, B. OVA/SEB and bZnO, and C. OVA/SEB and nZnO treated skin.** Scale bar 100 μm. Click here for file

Additional file 3: **F4/80 stained skin of A. OVA/SEB, B. OVA/SEB and bZnO, and C. OVA/SEB and nZnO treated skin.** Scale bar 100 μm. Click here for file

Additional file 4: **Antibody levels in mice sera after repeated topical application of vehicle, different sized ZnO, and OVA/SEB with or without particle application.** A. Total IgG2a values are showed as ratios compared to PBS-treated control and whereas B. OVA-specific IgG2a levels are showed as optical values measured at 405 nm. The columns and error bars represent means ± SEM (n=8 mice /group). **P<0.01. Click here for file

Additional file 5: The physical identity of ZnO particles used in the study. Click here for file

Additional file 1: **Murine model of AD used in this study.** In this model, the back skin of mice was shaved and tape stripped one to four times, mimicking skin injury inflicted by scratching in patients with AD. 100 μl of saline or a mixture of OVA and SEB in saline was placed on 1 cm2 patch of sterile gauze which was secured to the skin with a transparent bioocclusive dressing (A). Each mouse had a total of five exposures to the patch at the same site. Exposures were separated by two-week interval and ZnO materials were applied during the second sensitization week. Skin, blood and draining lymph nodes were collected for different analyses (B). Click here for file
