# Supplementary material for: Topically applied ZnO nanoparticles suppress allergen induced skin inflammation but induce vigorous IgE production in the atopic dermatitis mouse model
Source: Part Fibre Toxicol. 2014 Aug 14;11:38. doi: 10.1186/s12989-014-0038-4 (PMC4237966; doi:10.1186/s12989-014-0038-4)
Supplement: Additional file 2: — CD3, CD4 and CD8 stained skin of A. OVA/SEB, B. OVA/SEB and bZnO, and C. OVA/SEB and nZnO treated skin. Scale bar 100 μm. [file s12989-014-0038-4-S2.pdf]

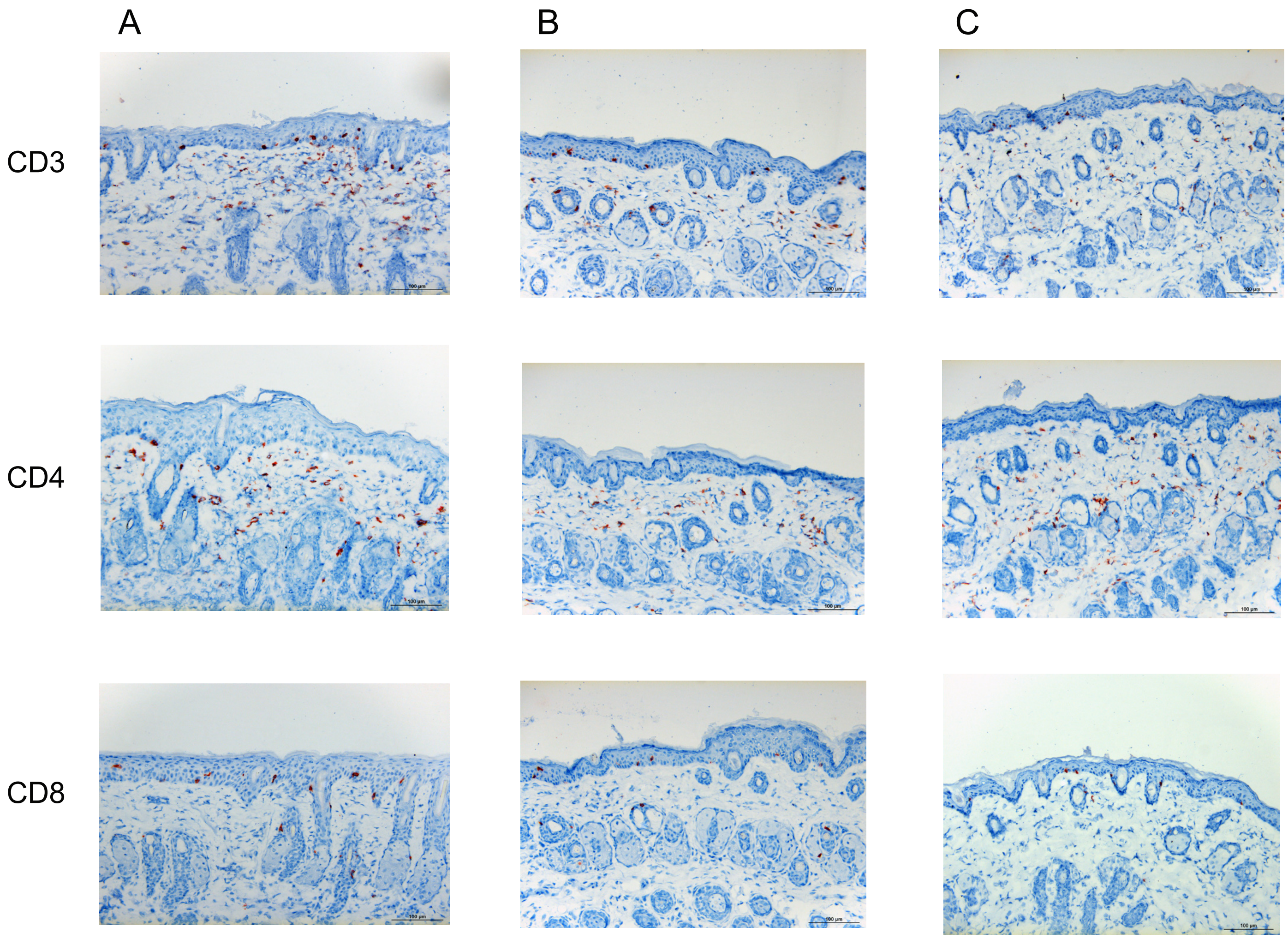

Additional file 2. CD3, CD4 and CD8 stained skin of A. OVA/SEB, B. OVA/SEB and bZnO, and C. OVA/SEB and nZnO treated skin. Scale bar 100 µm.
