# Supplementary material for: Topically applied ZnO nanoparticles suppress allergen induced skin inflammation but induce vigorous IgE production in the atopic dermatitis mouse model
Source: Part Fibre Toxicol. 2014 Aug 14;11:38. doi: 10.1186/s12989-014-0038-4 (PMC4237966; doi:10.1186/s12989-014-0038-4)
Supplement: Additional file 4: — Antibody levels in mice sera after repeated topical application of vehicle, different sized ZnO, and OVA/SEB with or without particle application. A. Total IgG2a values are showed as ratios compared to PBS-treated control and whereas B. OVA-specific IgG2a levels are showed as optical values measured at 405 nm. The columns and error bars represent means ± SEM (n=8 mice /group). **P<0.01. [file s12989-014-0038-4-S4.pdf]

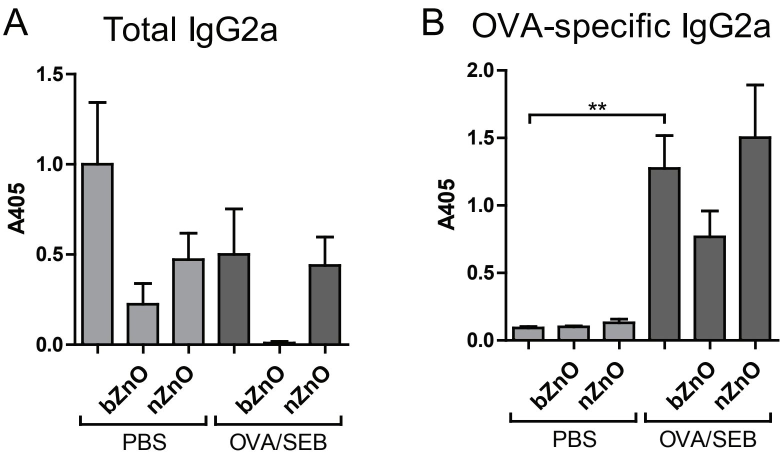

Additional file 4. Antibody levels in mice sera after repeated topical application of vehicle, different sized ZnO, and OVA/SEB with or without particle application. A. Total IgG2a values are showed as ratios compared to PBS-treated control and whereas B. OVA-specific IgG2a levels are showed as optical values measured at 405 nm. The columns and error bars represent means ± SEM (n=8 mice /group). ***P*<0.01.
