# Supplementary material for: Topically applied ZnO nanoparticles suppress allergen induced skin inflammation but induce vigorous IgE production in the atopic dermatitis mouse model
Source: Part Fibre Toxicol. 2014 Aug 14;11:38. doi: 10.1186/s12989-014-0038-4 (PMC4237966; doi:10.1186/s12989-014-0038-4)
Supplement: Additional file 5: — The physical identity of ZnO particles used in the study. [file s12989-014-0038-4-S5.pdf]

	**bZnO**	**nZnO**
Supplier	Camden-Grey Essential Oils, Inc	Nanostructured & Amorphous Materials, Inc
Product nr	-	5810MR
Size	240 nm (by vendor)	20 nm
Elements	Zn, O, residues of Fe	Zn, O
TEM morphology	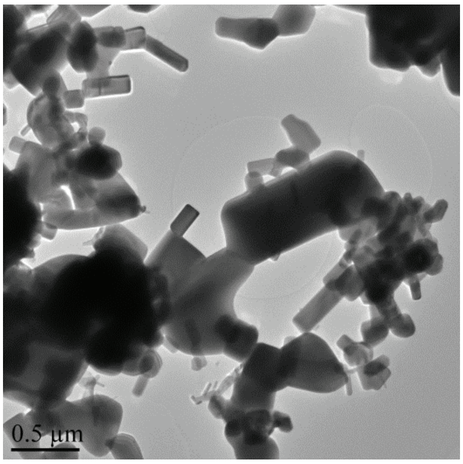	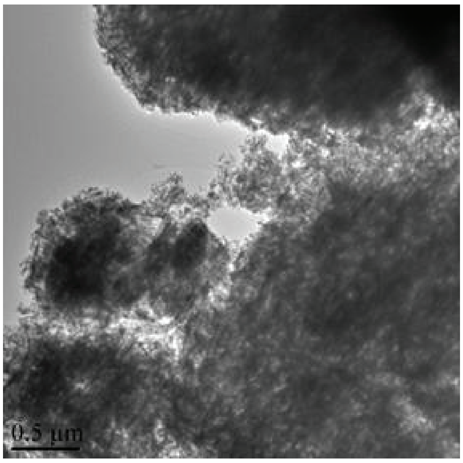

Additional file 5. The physical identity of ZnO particles used in the study.
