# Supplementary material for: Topically applied ZnO nanoparticles suppress allergen induced skin inflammation but induce vigorous IgE production in the atopic dermatitis mouse model
Source: Part Fibre Toxicol. 2014 Aug 14;11:38. doi: 10.1186/s12989-014-0038-4 (PMC4237966; doi:10.1186/s12989-014-0038-4)
Supplement: Additional file 1: — Murine model of AD used in this study. In this model, the back skin of mice was shaved and tape stripped one to four times, mimicking skin injury inflicted by scratching in patients with AD. 100 μl of saline or a mixture of OVA and SEB in saline was placed on 1 cm2 patch of sterile gauze which was secured to the skin with a transparent bioocclusive dressing (A). Each mouse had a total of five exposures to the patch at the same site. Exposures were separated by two-week interval and ZnO materials were applied during the second sensitization week. Skin, blood and draining lymph nodes were collected for different analyses (B). [file s12989-014-0038-4-S1.pdf]

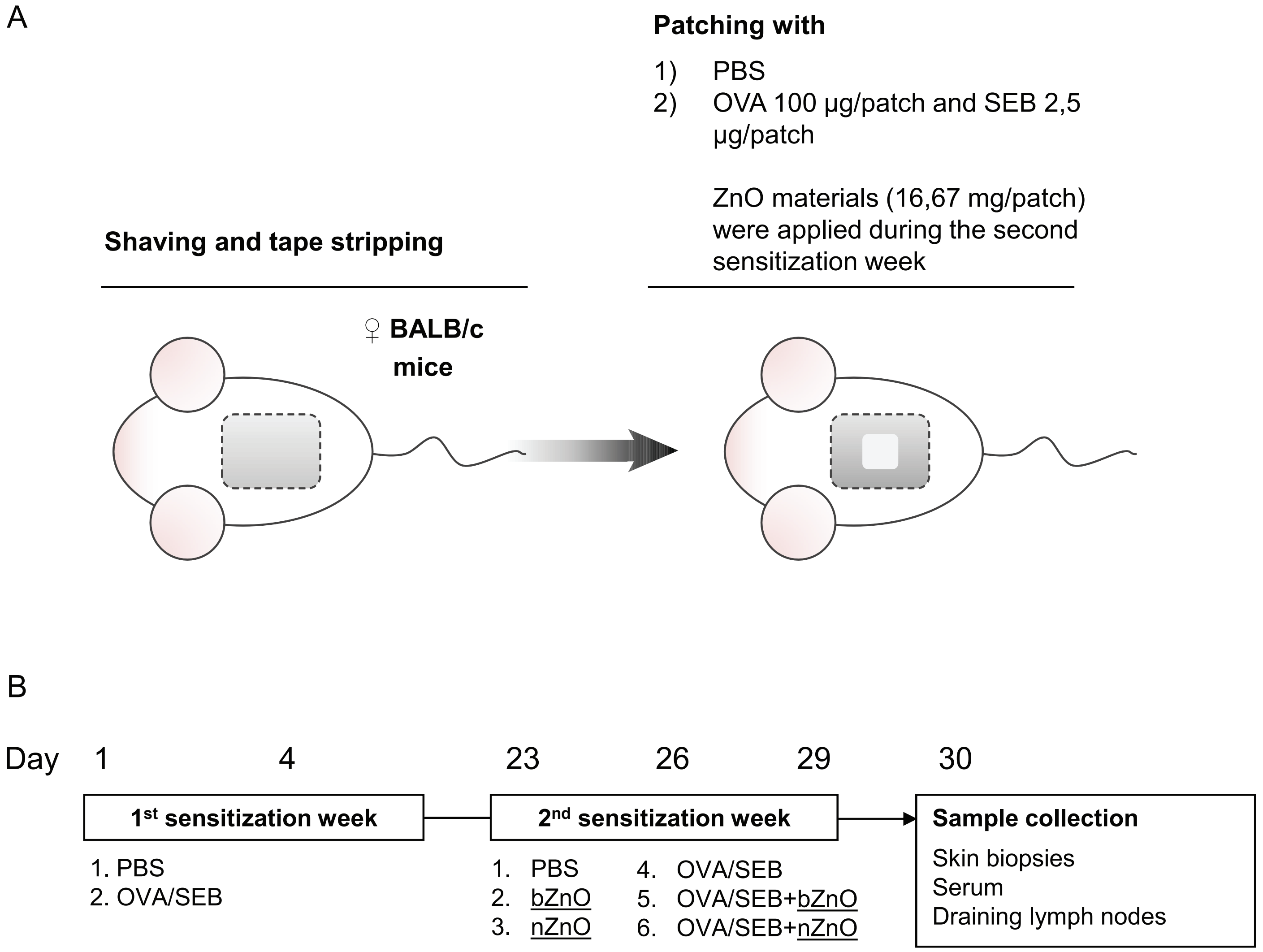

Additional file 1. Murine model of AD used in this study. In this model, the back skin of mice was shaved and tape stripped one to four times, mimicking skin injury inflicted by scratching in patients with AD. 100 µl of saline or a mixture of OVA and SEB in saline was placed on 1 cm^2 patch of sterile gauze which was secured to the skin with a transparent biocclusive dressing (A). Each mouse had a total of five exposures to the patch at the same site. Exposures were separated by two-week interval and ZnO materials were applied during the second sensitization week. Skin, blood and draining lymph nodes were collected for different analyses (B).
